# Severe Neurodevelopmental Disorder due to Klinefelter Syndrome and *CACNA1C* Variant: A Case Report

**DOI:** 10.1155/crpe/2844167

**Published:** 2025-11-19

**Authors:** Imen El Kamel El Lebbi, Séverine Bacrot, Myrtille Spentchian, Agnès Taillandier, Sophie Brisset, Geoffroy Delplancq

**Affiliations:** ^1^Laboratoire de Génétique Biologique, CHU Besançon, Université de Franche-Comté, Besançon, France; ^2^Constitutional Genetics Unit, Versailles Hospital, Le Chesnay, France

**Keywords:** aneuploidy, CACNA1C, double-hit, exome sequencing, karyotype, Klinefelter syndrome

## Abstract

Klinefelter syndrome (KS) is a common sex chromosome aneuploidy characterized by tall stature, hypogonadism, and learning disabilities. However, the severity of clinical presentation can vary significantly among individuals. We report a 13-year-old male patient adopted from Colombia who was diagnosed with KS at age 5.5 due to learning and behavioral difficulties. Despite the typical KS features, his clinical presentation was unusually severe, including significant developmental delay, behavioral issues, and physical abnormalities. Due to the uncommon severe presentation of KS in our patient, exome sequencing (ES) was performed, revealing a de novo heterozygous frameshift likely pathogenic variant in *CACNA1C* (c.2662del p.[Arg888Glyfs^∗^18]). *CACNA1C* pathogenic variants are associated with several phenotypes, including neurodevelopmental disorder with hypotonia, language delay, and skeletal defects with or without seizures. This case emphasizes the importance of considering additional genetic investigations in KS patients with atypically severe symptoms. Such an approach can identify secondary genetic events that contribute to the phenotype, guiding a more comprehensive clinical management and allowing a more precise genetic counseling.

## 1. Introduction

First described in 1942 [1], Klinefelter syndrome (KS) is a common sex chromosome aneuploidy characterized by the presence of an extra X chromosome in males (47, XXY), leading to a spectrum of clinical manifestations. These often include tall stature, hypogonadism, infertility, learning disabilities, verbal intellectual quotient (IQ) frequently below normal, and social difficulties. The severity and presentation of symptoms can vary significantly among individuals [2]. Cases can be incidentally diagnosed prenatally, in childhood or in adult life. A Danish registry study showed that less than 10% of KS are diagnosed before puberty [3].

*CACNA1C* (MIM 114205) encodes the alpha-1 subunit of a voltage-dependent L-type calcium channel that is broadly expressed in human heart, brain, smooth muscle, and endocrine tissue [4]. Heterozygous pathogenic variants in the *CACNA1C* gene are associated with several autosomal dominant phenotypes. Specific heterozygous gain-of-function (GOF) variants are associated with Timothy syndrome (MIM #601005), a severe, multisystem disorder characterized by autism, epilepsy, long-QT syndrome and other neuropsychiatric conditions [5]. GOF variants have also been reported in association with nonsyndromic long-QT syndrome (MIM #618447) [6]. Loss of function (LOF) leads to a neurodevelopmental disorder with hypotonia, language delay, and skeletal defects with or without seizures (MIM #620029) [7]. LOF variants have also been associated with the Brugada syndrome (MIM #611875) [8].

We report a 13-year-old boy with KS and a likely pathogenic *CACNA1C* variant, highlighting the combined impact of these genetic abnormalities on his phenotype and the importance of further genetic investigations in complex KS cases.

## 2. Clinical Report

At age 5,5, a male boy adopted from Colombia at 18 months, was referred for a genetic evaluation due to learning difficulties and behavioral problems. No data of the familial history were available. The patient was born at term with a birth weight of 2–2.5 kg. He began walking at 16 months. Speech development was significantly delayed, although his hearing was normal. He repeated a year in preschool and received weekly support from an assistant. At the first examination, the patient's weight was 22.8 kg (+1.5 SD), height was 119.7 cm (+1.5 SD), and cranial circumference was 51.5 cm (+1 SD). He exhibited gingival hypertrophy, hyperlaxity of fingers, and an elongated face. He began speech and psychomotor therapy. First, genetic explorations were performed at that time including standard karyotype, microarray-based comparative genomic hybridization (CGH-array), and Fragile X syndrome.

At age 9.9, his height was +2 SD, indicating an increased growth velocity. He was managed for multiple allergies, including dust mites and cat fur, and showed early signs of puberty. A gingival curettage was decided to assist with oral occlusion and to prepare for future dental appliances in the coming years. He was enrolled in a specialized education program due to severe behavioral issues and significant learning difficulties, especially in mathematics. A WISC-V assessment revealed an IQ of 57.

At age 11.9, he was treated with melatonin for sleeping disturbance. His height was 133, 3 cm (+3.1 SD) and his weight was 44.5 kg (+1, 5 SD). The pubertal stage was A1, P3. Signs of early testicular insufficiency were observed, including elevated levels of luteinizing hormone (LH) and follicle-stimulating hormone (FSH). Small testes and gynecomastia were noted.

**Figure 1:**
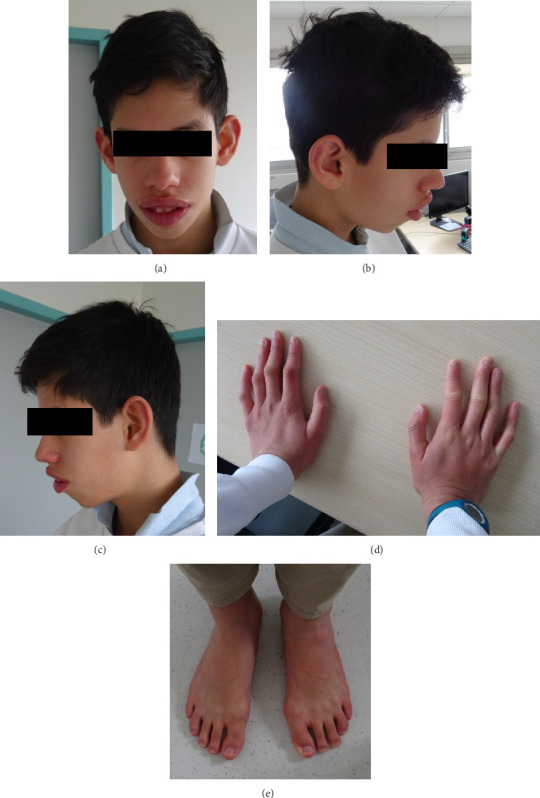
Photographs of the patient at 13 years old of life ((a) face; (b) right profile; (c) left profile; (d) hands; (e) feet). (a–c) Elongated face and thick and anteverted lips. (d) Long fingers. (e) No anomaly of the feet or toes.

At age 13, he was 172 cm tall (+2, 5 SD) and weighed 53.5 kg (+1, 5 SD). Persistent gingival hypertrophy, dental misalignment, and large and protruding ears were observed (Figure 1(a)). He has a long face and thick and anteverted lips (Figures 1(a), 1(b), and 1(c)), and long fingers (Figure 1(d)). No abnormalities were noted in feet or toes (Figure 1(e)). His cardiac evaluation including ECG was normal. Treatment with Risperdal was initiated but discontinued due to limited effectiveness and a tendency to cause lethargy. Due to a more severe neurological phenotype than would be expected from KS alone, it was decided to pursue further genetic investigations with exome solo sequencing (ES).

### 2.1. Genetic Investigations

**Figure 2:**
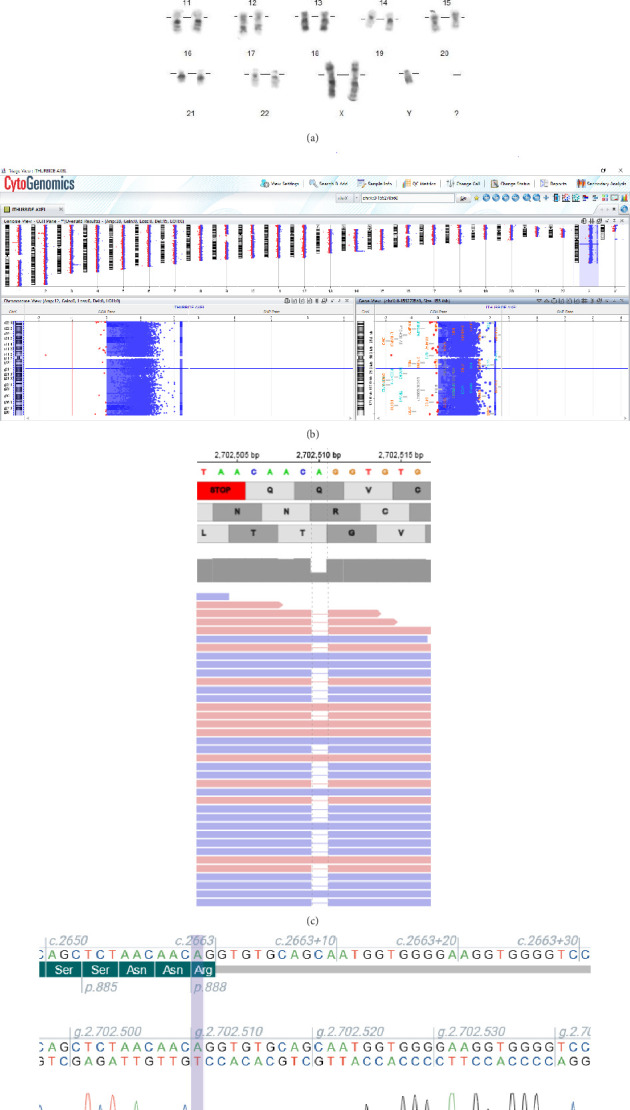
Conventional cytogenetic and molecular genetic analyses of the patient. (a) Conventional resolution G-banding karyotype at 550 bands shows an extra chromosome X (47, XXY). (b) Screen capture of the cytogenomics view of the 180K CGH-array, showing a duplication of the entire X chromosome. CGH-array: arr(1–22)x2, (X)x2, (Y)x1. (c) IGV (integrative genomics viewer) capture of the *CACNA1C* heterozygous variant. (d) Sanger sequencing capture of the *CACNA1C* heterozygous variant, confirming the diagnosis viewed via the software Archigene (https://www.archigene.com/en/).

Genetic analyses were performed after obtaining informed consent. After the first counseling (5,5 years old), a conventional G-banded karyotype (550 bands) was performed and identified an extra X chromosome leading to the diagnosis of KS (Figure 2(a)). CGH-array was performed on genomic DNA, extracted from peripheral blood by a conventional method, using Agilent Human Genome CGH 180K oligonucleotide arrays (Agilent, Santa Clara, CA, USA; https//:www.agilent.com) following manufacturer's protocols. The array was analyzed with the CytoGenomics 3.0.4.1 software and confirmed the extra X chromosome without additional chromosomal imbalances (Figure 2(b)). The test for Fragile X syndrome by CGG triplet expansion came back normal.

After the second genetic counseling (13 years old), given the severity of the clinical features, ES was performed. The enrichment of exomic regions was performed using the “TWIST Comprehensive Exome Panel” (TWIST) followed by a paired-end sequencing reaction (2 × 100 bp) on the NextSeq1000 (Illumina). Bioinformatics processing of the data was carried out using the SeqOne platform (https://seqone.com/). The diagnostic interpretation of the filtered variants was performed according to the American College of Medical Genetics and Genomics (ACMG). It reveals a de novo heterozygous frameshift variant in the Exon 19 (/47) of the *CACNA1C* gene: NC_000012.11:g.2702510del, NM_000719.7:c.2662del, NP_000710.5:p.(Arg888Glyfs^∗^18), that was never reported either in gnomAD, LOVD, or ClinVar. SpliceAI [9] predicts a gain of a donor cryptic site 24 bases downstream (score: 0.72) without loss of the physiological donor splice site. This indicates that it is indeed a truncating variant. The gene is predicted to be intolerant to LOF variants (pLI = 1; observed/expected score of 0.13–90% and confidence interval [CI] of 0.09–0.19 in gnomAD V4.1.0). According to the ACMG recommendations, this variant has been classified as likely pathogenic (class 4).

Figure 2(c) shows the IGV capture of the *CACNA1C* variant identified by ES, and Figure 2(d) shows the Sanger sequencing confirmation of the variant.

## 3. Discussion

We report here a patient characterized by severe learning difficulties, significant behavioral issues, and a low IQ. The severe phenotype was more pronounced than typically expected in KS. Indeed, the average IQ in KS is lower than the general population, typically in the 85–90 range, but profound intellectual disability (ID) is not a characteristic feature. This led us to investigate additional genetic causes. The identification of a likely pathogenic variant in *CACNA1C* confirmed our suspicion of a “double diagnosis.” Table 1 compares the key clinical features of the patient (e.g., developmental delay [DD], IQ, behavioral issues, and facial features) with the typical features of KS alone and CACNA1C-related neurodevelopmental disorder based on Rodan et al. [7].

As traditionally described, patients with KS have tall stature, small testes, gynecomastia in late puberty, gynoid aspect of hips (broad hips), sparse body hair, signs of androgen deficiency and low serum testosterone coupled with elevated gonadotropins, and finally azoospermia, oligospermia with hyalinization and fibrosis of the seminiferous tubules [10]. Neurocognitive profiles of KS patients typically include deficits in executive function, attention, and processing speed, with particular difficulties in verbal skills and working memory, leading to significant challenges in academic and social settings. Despite these typical features, the severity of the neurodevelopmental phenotype can vary significantly. Numerous individuals with KS display no cognitive or behavioral deficits, highlighting the variability of this phenotype [11]. Most of the patients remain underdiagnosed. Psychiatric disorders such as depression, anxiety, and schizophrenia are also more prevalent in individuals with KS compared to the general population [11]. Probably, the phenotype depends on the severity of the expression of the genetic defect (mosaicism or not), androgen deficiency, and androgen receptor sensitivity. The more the genetic expression, androgen deficiency, and androgen receptor sensitivity are worse, the more the phenotype will be severe [12]. Copy-number variant might be also considered as an additional independent genetic factor for ID and DD for patients with KS and neurodevelopmental disorder [13].

*CACNA1C* encodes the alpha-1C subunit of the L-type calcium channel, which is critical for the normal function of many tissues, including the brain and the heart. It has been implicated in the development of the serotonergic system [14]. The first identified *CACNA1C*-related disorder, referred to as Timothy syndrome, consists of the combination of prolonged QT interval, autism, and cardiovascular malformation with syndactyly of the fingers and toes. Infrequent findings also include developmental and speech delay, seizures, and recurrent infections. With increased availability of molecular genetic testing, a wider spectrum of pathogenic variants and clinical findings associated with *CACNA1C*-related disorders has been recognized such as neurodevelopmental abnormalities and epilepsy, in the absence of classic features of Timothy syndrome or long-QT syndrome [15]. Timothy syndrome is caused by CACNA1C GOF. Conversely, truncation/deletion of *CACNA1C* has previously been reported in association with DD/ID, and it has been suggested that *CACNA1C* is intolerant to LOF. It seems that the more severe neurodevelopmental and epilepsy phenotype in patients with missense, predicted LOF variants compared to those with whole-gene/exonic deletions would better support a dominant-negative effect.

Given the compounded effects of KS and the *CACNA1C* variant, the patient's management involved multidisciplinary care, including endocrinology, neurology, cardiology, orthopedics, and specialized educational support. This “double diagnosis” can explain the severity of the neurological phenotype of our patient.

This case highlights the importance of considering additional genetic investigations in KS patients with atypically severe symptoms, especially DD/ID. The identification of a secondary pathogenic variant in *CACNA1C* provides valuable insights into the patient's complex phenotype and guides a more comprehensive clinical management approach. It is also important for genetic counseling and antenatal diagnosis. As in the general population, a causative event should be looked for in individuals with KS showing DD or ID. It also underlines that despite high throughput and methylome sequencing, conventional cytogenetics exploration can lead to a diagnosis in cases of DD/ID.

## Figures and Tables

**Table 1:** Phenotype comparison of the patient, Klinefelter syndrome alone, and *CACNA1C*-related neurodevelopmental disorder based on Rodan et al. [7].

	Case report	Klinefelter syndrome	*CACNA1C*-related neurodevelopmental disorder [7]
Motor delay	+	+/−	+ (17/25)
Language delay	+	+/−	+ (22/25)
Hypotonia	+	+/−	+ (15/25)
Cognition	IQ = 57	IQ is typically in the 85–90 range	From normal to severe ID
Seizures	−	−	+/− (12/25)
Behavioral issues	Severe	Social difficulties	− ASD (6/25); ADHD (3/25)
Dysmorphy	Long face and thick and anteverted lips, gingival hypertrophy, dental misalignment, large and protruding ears, long fingers	Sparse body and facial hair, broader hips, gynecomastia, reduced muscle mass	+/− (13/25) Minor, not specific. Large ears, mildly broad thumbs, hypoplastic 5th fingernails, broad halluces, and hypoplastic fifth toes, pes plano valgus, clubfoot in infancy, and small appearing feet with prominent heels, adducted thumb, and camptodactyly and kyphoscoliosis
Tall stature	+	+	−
Hypogonadism	+	+	−

## Data Availability

Data are available on demand.
